# Scarcity Mindset Neuro Network Decoding With Reward: A Tree-Based Model and Functional Near-Infrared Spectroscopy Study

**DOI:** 10.3389/fnhum.2021.736415

**Published:** 2021-11-24

**Authors:** Xiaowei Jiang, Chenghao Zhou, Na Ao, Wenke Gu, Jingyi Li, Yanan Chen

**Affiliations:** ^1^Institute of Psychology and Behavior, Henan University, Kaifeng, China; ^2^Institute of Cognition, Brain and Health, Henan University, Kaifeng, China; ^3^Department of Bioengineering, University of Pennsylvania, Philadelphia, PA, United States

**Keywords:** scarcity, reward, fNIRS, functional connectivity, prefrontal cortex

## Abstract

Resource scarcity imposes challenging demands on the human cognitive system. Insufficient resources cause the scarcity mindset to affect cognitive performance, while reward enhances cognitive function. Here, we examined how reward and scarcity simultaneously contribute to cognitive performance. Experimental manipulation to induce a polar scarcity mindset and reward conditions within participants under functional near-infrared spectroscopy (fNIRS) recording was implemented to explore the mechanism underlying the scarcity mindset and reward in terms of behavior and neurocognition. Participants showed decreased functional connectivity from the dorsolateral prefrontal cortex (DLPFC) to the ventrolateral prefrontal cortex (VLPFC) with a scarcity mindset, a region often implicated in cognitive control. Moreover, under reward conditions, the brain activation of the maximum total Hb bold signal was mainly located in the left hemisphere [channels 1, 3, and 4, left ventrolateral prefrontal cortex (L-VLPFC) and channel 6, left dorsolateral prefrontal cortex (L-DLPFC)], and there was also significant brain activation of the right dorsolateral prefrontal cortex (R-DLPFC) in the right hemisphere (channel 17). Furthermore, these data indicate the underlying neural changes of the scarcity mentality and demonstrate that brain activities may underlie reward processing. Additionally, the base-tree machine learning model was trained to detect the mechanism of reward function in the prefrontal cortex (PFC). According to SHapley Additive exPlanations (SHAP), channel 8 contributed the most important effect, as well as demonstrating a high-level interrelationship with other channels.

## Introduction

In the reality of social life, there are various forms of scarcity. These include the scarcity of capital sources, such as unemployment; lack of social contact and human ties, leading to social loneliness; or a dieter facing hunger and calorie loss. Additionally, scarcity can affect a group that has to make decisions with limited resources in a limited time to face challenges. Growing scarcity has been highlighted during the COVID-19 pandemic.

Cognitive performance is damaged during a scarcity mindset (Shah et al., 2012; Mani et al., 2013; Vohs, 2013; Nash et al., 2020). For instance, Mani et al. (2013) proposed that poverty decreased cognitive task performance, working memory, and logical thinking. On the other hand, recent work suggests that potential reward-driven motivation enhances executive control mechanisms that help prioritize goal-relevant information, leading to improved performance across perceptual and cognitive tasks (Pessoa, 2013; Botvinick and Braver, 2015). For instance, rewards boosted performance during working memory (Savine et al., 2010) and response conflict (Krebs et al., 2011) tasks.

Notably, the impacts of scarcity and reward on cognition have been mainly studied independent of one another. Therefore, our current understanding of how reward and scarcity simultaneously contribute to cognitive performance is incomplete. To address this gap, the present study investigated how scarcity and reward interact with one another during a modified monetary incentive delay (MID) task. Understanding these interactions is essential because many real-life situations involve both poverty and reward dimensions. Recent estimates show that approximately 20% of the world’s population is in poverty. Governments and organizations channel substantial money and resources into programs for the poor. Consequently, probing cognitive mechanisms involves the joint processing of scarcity and reward signals, and understanding interactions between reward and scarcity during cognition might be of potential benefit in anti-poverty projects sponsored by governments.

### Scarcity

Scarcity has a variety of negative consequences. For example, poor economic prospects have been associated with higher levels of stress, anxiety, depression, and suicide.

Recent studies focus on the cognitive consequences of the scarcity mindset caused by insufficient resources. For example, scarcity will impede human cognitive capacity (Mani et al., 2013) and generate a nearsighted strategy in decision-making by ignoring possible future issues (Shah et al., 2012). Mental resources are consumed by poverty-related concerns, leaving less for other tasks, which reduces fluid intelligence and the ability to exercise cognitive control (Mani et al., 2013). Individuals pay attention to emergency needs due to insufficient resources and trade off these needs (Shah et al., 2015). These cognition and behavior consequences are severe and result in poor decision-making and behavior (e.g., poor time planning and financial planning), thus intensely aggravating the condition of insufficient resources.

In summary, this situation can be explained by the cognitive constraint hypothesis (Howes et al., 2004). Individuals with high poverty levels feel insecure about the environment, anxious about money and the future, resentful about losing money, dissatisfied with money, and more emotional attachment to money. These negative emotional responses encourage individuals to pursue money more actively or to be more careful not to lose money. Recently, some research has revealed that exposure to a reward or reward demand activates a general reward system that prompts people to seek anything rewarding (Van den Bergh et al., 2008; Wadhwa et al., 2008). Thus, such a mechanism contributes to the self-regulatory failure of individuals in poverty, as their continued financial deprivation makes them more sensitive to reward cues.

### Reward

Reward facilitates perceptual processing and improved cognitive performance across a diverse set of tasks (Pessoa, 2010, 2013; Aarts et al., 2011; Botvinick and Braver, 2015; Padmala et al., 2017). For instance, in MID paradigms (Knutson et al., 2000; Knutson and Cooper, 2005), a prior cue indicating a monetary reward condition for an upcoming trial typically signals a reward expectation: an additional monetary reward is possible for the individuals if they perform fast and accurately, whereas no reward is offered in the no-incentive condition. Similarly, a potential reward has been shown to reduce switching costs in task-switching paradigms (Savine et al., 2010), decrease stop-signal reaction times during response inhibition tasks (Boehler et al., 2012), and increase postconflict control in flanker tasks (Braem et al., 2012). Reward expectations improve the allocation of attention to target stimuli and decrease attention to distractors, leading to improved behavioral performance (Chelazzi et al., 2013; Krebs and Woldorff, 2017).

Evidence from behavioral, event-related potentials, and neuroimaging measures suggests that the MID (Knutson et al., 2000) task is a widely used paradigm for examining reward processing (MTAC et al., 2018). An fMRI study (Ballard et al., 2011) found that the dorsolateral prefrontal cortex (DLPFC) is the only entrance to this reward information network, converting reward information to reward motivation. The expected reward availability leads to activation of the ventral tegmental area (VTA) only through its effect on the DLPFC. Thus, reward anticipation directly increases activation in the DLPFC, whereas it only indirectly affects the VTA and the nucleus accumbens (NACC) by enhancing weak or inactive pathways inherent in the DLPFC (Ballard et al., 2011). Overall, the DLPFC integrates reward representations and transmits them to the mesolimbic and dopamine systems to initiate motivational behavior.

### Current Study

Despite the far-reaching psychological effects mentioned above, the impact of the scarcity mindset and reward expectation on basic neurocognitive mechanisms remains unclear. Here, we attempt to directly examine the relationship between the scarcity mindset and reward expectation with functional near-infrared spectroscopy (fNIRS).

Investigating how the scarcity mindset and reward simultaneously contribute to behavioral performance and neurocognitive activation in a laboratory setting is a challenging endeavor – paradigms include, for example, limiting shots in an experimental shooting game (Shah et al., 2012), consumption (Roux et al., 2012), hunger due to food restriction (Aarøe and Petersen, 2013; Xu et al., 2015), exposure to faux articles about the economic recession (Griskevicius et al., 2013), and current income level (Mani et al., 2013). However, these manipulations are not always dependent on the current state of scarcity. They may be confused with past life experiences by individuals or rely on task-required skills. Currently, the mechanism underlying cognitive change due to scarcity is not clear. One possible explanation is that the state of resource scarcity causes individuals to use more of their attention resources, resulting in more urgent resource demands and leading to a stronger focus on the task at hand. Such attention often leads to neglect of other potentially important information (Tomm and Zhao, 2018).

This study examines the interactive effect of scarcity and reward on neurocognitive processes and can help shed light on the mechanisms involved in these broader psychological consequences. Based on studies of cognitive function defects in poverty (Shah et al., 2012; Mani et al., 2013; Vohs, 2013), we tentatively predicted that people with a scarcity mindset might show poorer performance than those with an abundance mindset. Furthermore, we hypothesized that the processing of a reward cue would lead to increased responses in the left prefrontal cortex (PFC) (Cho et al., 2016), as well as an interrelationship within the PFC. Therefore, to explore the interactive contribution of channels and features of importance, this study trained some tree-based machine learning models to explain them. As previous research did not distinctly manipulate the scarcity mindset in a laboratory setting, and the existing literature on the interaction of scarcity and reward is relatively sparse, no specific prediction was formed in this regard.

## Materials and Methods

### Participants

Forty freshman-and-sophomore-year subjects with no history of neurological or psychiatric disease took part in our study (males = 23, female = 17, mean age = 20 ± 1.2 SD). They were undergraduates studying at Henan University and signing the informed consent. Because of the recording system’s error, we lose one subject’s fNIRS data, and his behavior data was intact, so we included it in the analysis. Eight subjects did not finish the experiment since discomfort, or stopping by researchers as the bad quality of fNIRS channels occurring during the experiment; five subjects were excluded for their poor performance (behavioral accuracy of any condition <0.5), and the other two subject fNIRS data were removed because their fNIRS records had too much noise (their behavior data was intact and preserved). Finally, this study remains 25 valid fNIRS data and 27 valid behavioral data. After the experiment, all subjects got remuneration.

### Procedure

The experiment examined scarcity and reward conditions and the neural processes associated with them. This study modified the MID experimental task paradigm by manipulating the scarcity mindset and reward conditions, and PTB3 and MATLAB R2020a were used to analyze the results. The experiment consisted of two modules, corresponding to two different amounts of start-up capital (0 yuan or 1000 yuan) representing launch conditions of scarcity and two reward conditions. The pseudorandom method was used to balance start-up funding conditions. If the number of participants was odd, the first start-up capital fund was 0 yuan, and the second start-up capital was 1000 yuan. If the number of participants was even, the first start-up capital fund was 1000 yuan, and the second start-up capital fund was 0 yuan. Participants were scanned by fNIRS while completing the task. Each module contained 80 trials, of which 40 were randomly presented for each of the 2 reward conditions.

**FIGURE 1:**
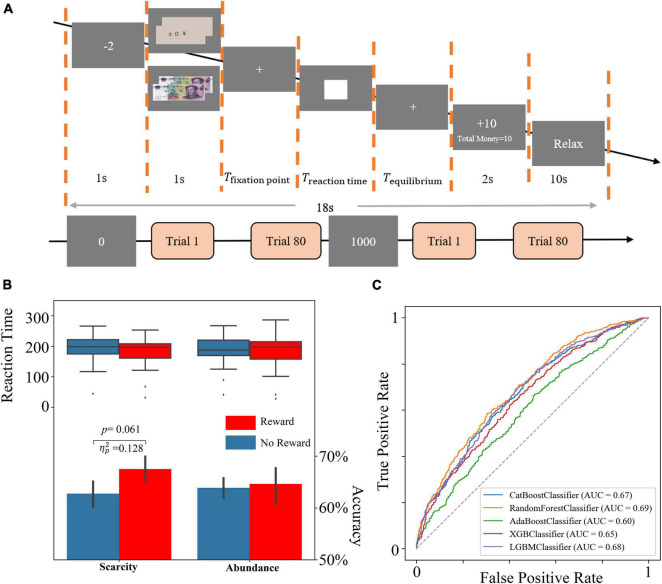
**(A)** The flow graph. **(B)** The behavior results. **(C)** The ROC curves of all tree-based models.

The participants were shown the amount of money they currently had (0 or 1000 yuan) before each module as a trigger for a scarcity mindset and then randomly presented each trial. In each trial, participants were first told that they had been charged $2 as the round cost and were then presented with a reward condition (either ¥0 or ¥10) as the incentive condition. Then, the participants were presented with a fixation point that ranged between 1 and 2 s randomly to prevent them from guessing about subsequent tasks. The subjects were next shown a white rectangular square as the task image for T seconds. Participants were required to press the space within T seconds. Otherwise, the timeout was judged to have failed. T was initially 200 ms; if the current total accuracy was less than 66%, T was multiplied by 110% until T was greater than 320 ms. If the current total accuracy was more significant than 66%, T was multiplied by 90% until T was less than 80 ms. The subjects were then presented with 2-s feedback that informed them of their current round of gains, as well as their current total amount of money. Next, the subject was provided with a variable waiting time *T*_*equilibrium*_ to balance the entire test time, where *T*_*equilibrium*_ was shown below. If no key was pressed at the specified time, *T*_*reactiontime*_ was T. At the end of the 10-s break, one trial ended, and the next began. The flow chart is shown in Figure 1A.


(1) $$T_{\text{equilibrium}}=4-T_{\mathrm{fixation}\,\mathrm{point}}-T_{\mathrm{reaction}\,\mathrm{time}}$$

### Behavioral Experimental Data Acquisition and Analysis

This study recorded the reaction time and accuracy and calculated each participant’s average reaction time and accuracy under the four conditions at the end of the experiment, used for subsequent calculation. Based on all participants’ data, the main effects and the interaction effects of the two main effects were examined in SPSS25 using repeated measures analysis of variance.

### Functional Near-Infrared Spectroscopy Data Acquisition and Analysis

**FIGURE 2:**
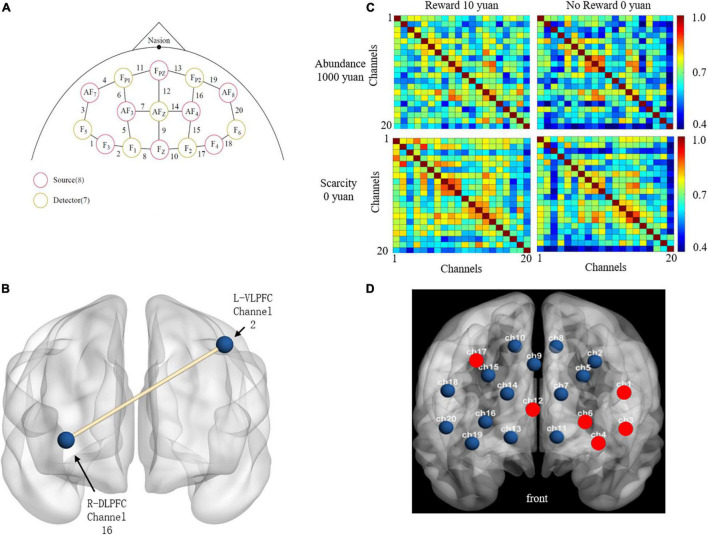
**(A)** It shows that the position of the sources, detections, and channels (Yuan et al., 2020). **(B)** The abridged general view of the functional connectivity between channel 16 in R-DLPFC and channel 2 in L-VLPFC. **(C)** The functional connectivities between reward conditions and sufficient resources. **(D)** The activated channels were shown as red point.

The absorptivity of oxy-Hb, deoxy-Hb, and total-Hb was measured using a continuous-wave system (NIRX Scout 32 × 32, United States) and an 8 × 7 probe set covering the PFC with a total of 20 channels consisting of 8 light transmitters and 7 detectors. The fNIRS system in this study uses two different wavelengths (785 and 830 nm), and the frequency is adjusted according to wavelength and channel to avoid crosstalk. The distance of each probe is 30 mm, and the sampling rate of the device is 7.8125 Hz. The probe is mounted on the swimming cap according to the International 10–20 system and covers the PFC. This study focused on the PFC region to explore brain function. Figures 2A,D shows the fNIRS channel setup based on the 10–20 system (EEG).

This study set four ROI referred pilot study (Yuan et al., 2020), including the right ventrolateral prefrontal cortex (R-VLPFC) (17–20), right dorsolateral prefrontal cortex (R-DLPFC) (13–16), left dorsolateral prefrontal cortex (L-DLPFC) (5–7 and 11), and left ventrolateral prefrontal cortex (L-VLPFC) (1–4), shown in Table 1. The data were statistically analyzed using MATLAB R2020a software. Firstly, the raw optical signals were converted into oxy-Hb, deoxy-Hb, and total-Hb signals by the Beer–Lambert equation using Homer2 software, and the motion artifacts were removed. A bandpass filter filtered the frequency of 0.01 and 0.15 Hz to remove heart rate or other low-frequency interference and high-frequency noise. After that, functional connectivity was calculated by correlation coefficient (r)


(2) $$\text{r}\,\left(X,Y\right)=\frac{C\,o\,v\,\left(X,Y\right)}{\sqrt{V\,a\,r\,\left[X\right]\,V\,a\,r\,\left[Y\right]}}$$

where, *Cov*(*X*,*Y*) is the covariance of channel *X* and channel *Y*, *Var*[*X*] is the variance of channel *X*, *Var*[*Y*] is the variance of channel *X*. After calculating each of the participants’ average superposition data in different conditions of 40 trials, repeated ANOVA (rANOVA) was used to compare the max activation and functional connectivity. Due to multiple comparisons, false discovery rate (Benjamini and Yekutieli, 2005) was used to correct the *p*-values.

**TABLE 1:** Each channel located in ROI and rANOVA results.

ROI	Channel	Standard location			Sample analysis: condition (mean)	*F*(1,24)	$\eta_{p}^{2}$
		*x*	*y*	*z*			
L-VLPFC	1	0.4867	−0.4283	0.7614	R(1.94) > NR(1.47)	10.1171***	0.3166
L-VLPFC	2	0.3307	−0.3837	0.8622	R(1.67) > NR(1.43)	1.2582	0.0860
L-VLPFC	3	0.5360	−0.5621	0.6299	R(1.98) > NR(1.45)	9.6893***	0.3081
L-VLPFC	4	0.3670	−0.7381	0.5660	R(2.66) > NR(1.98)	11.1673***	0.3364
L-DLPFC	5	0.2799	−0.4936	0.8234	R(1.22) > NR(1.10)	2.8396	0.1379
L-DLPFC	6	0.2997	−0.7020	0.6460	R(1.90) > NR(1.47)	7.3596*	0.2583
L-DLPFC	7	0.2001	−0.6178	0.7605	R(1.19) > NR(1.01)	2.3739	0.1233
/	8	0.1171	−0.4085	0.9052	R(1.81) > NR(1.73)	0.2093	0.0480
/	9	0.0338	−0.5225	0.8520	R(1.77) > NR(1.46)	5.5874	0.2154
/	10	−0.0769	−0.4226	0.9031	R(2.17) > NR(1.83)	5.1768	0.2047
L-DLPFC	11	0.1680	−0.7997	0.5764	R(2.35) > NR(1.94)	4.3002	0.1809
/	12	0.0275	−0.7247	0.6886	R(1.36) > NR(0.96)	11.6184***	0.3446
R-DLPFC	13	−0.1005	−0.8202	0.5632	R(2.10) > NR(1.72)	3.2144	0.1494
R-DLPFC	14	−0.1366	−0.6321	0.7628	R(1.23) > NR(1.03)	2.6329	0.1315
R-DLPFC	15	0.2383	−0.5223	0.8188	R(1.37) > NR(1.15)	5.5833	0.2153
R-DLPFC	16	−0.2462	−0.7303	0.6372	R(1.75) > NR(1.45)	3.9624	0.1713
R-VLPFC	17	−0.2977	−0.4217	0.8565	R(2.15) > NR(1.68)	8.5510**	0.2847
R-VLPFC	18	−0.4766	−0.4575	0.7507	R(1.64) > NR(1.44)	1.9698	0.1101
R-VLPFC	19	−0.3138	−0.7739	0.5501	R(2.05) > NR(1.62)	5.5455	0.2143
R-VLPFC	20	−0.5150	−0.5995	0.6127	R(1.82) > NR(1.58)	1.1691	0.0829

ROIs labeled with “/” were not classified into any brain regions in this study. In simple effect analysis, R was 10 yuan reward, and NR was none reward. ***p < 0.001; **p < 0.01; *p < 0.05.

### Tree-Based Model

Common integrated tree-based models have two categories: one is the bagging model, such as random forest, which compares the results of each tree to obtain the final result by voting, and the result is subordinate to the minority and the majority (Barnett et al., 1989; Breiman, 2001). Each tree uses random sampling samples (bootstrap sampling) in the entire sample set and predictive variables (Breiman, 2001; Grömping, 2009). Such a processing method effectively forces the tree to produce instability and produces differences between trees to obtain better total fitting results (Barnett et al., 1989). Another type is the boosting model, which is a robust classifier composed of weak classifiers based on different weights, and weak classifiers are mutually dependent. Boosting models are commonly used in AdaBoost (Freund and Schapire, 1995), XGBoost (Chen and Guestrin, 2016), LightGBM (Ceperic and Baric, 2004), and CatBoost (Prokhorenkova et al., 2019).

In order to fit the dataset better, this study used these five different tree-based models (Random Forest, CatBoost, AdaBoost, XGBoost, and LightBoost) to compare, using all valid trials as samples and using each channel as a feature for prediction. In the classification model training, “reward” (or “1”) and “no reward” (or “0”) were set as predictive variables in this study. Each label has N samples


(3) $$N=2\,N_{\text{subjective}}\,N_{\mathrm{repeated}\,\mathrm{time}}$$

where *N*_subjective_ is 25 and *N*_repeated time_ is 40.

**FIGURE 3:**
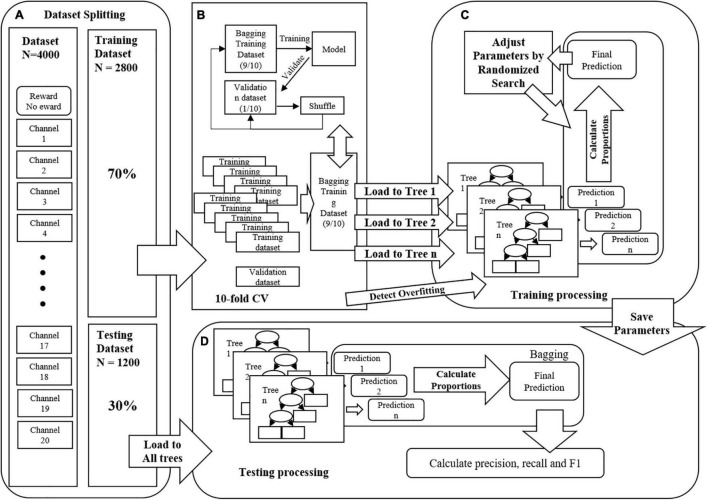
**(A)** The features were channels, the samples were 4000 firstly, then split into two classes, training dataset (70%, 2800) and testing dataset (30%, 1200), respectively. **(B)** The training dataset was split randomly into 10 sub-datasets as 10-fold cross-valid processing, 9 were bagged as training dataset, and 1 was validation dataset. **(C)** The processing of training model. **(D)** The processing of testing model.

This study hypothesizes that classification models can predict the difference between different reward conditions. Therefore, this study used all the data of two parts under two different scarcity mindset conditions simultaneously. The dataset is randomly shuffled and divided into two parts. The first part is the training dataset (70%, 2800 samples) to train the machine learning algorithm. The other part is the test dataset (30%, 1200 samples), which acts as an independent test set to validate the final model. This study used 10-fold cross-validation (10-fold CV) to establish the training dataset classification model. More precisely, selecting 90% training dataset randomly to train the model while the other 10% training dataset was used to verify it. All models use Gini Index as the entropy coefficient of the model. Gini Index could measure the variance. Higher Gini Index means the higher mis-classification. Accordingly, the random grid method was used to decease this variance as the searching method for the optimal parameters. The best performing model was selected as the interpreter model for subsequent analysis according to the training results. The training process is shown in Figure 3. The Gini coefficient was used to calculate the importance of the optimal model’s input features to obtain each feature’s importance. Finally, the results’ reliability was verified using the SHapley Additive exPlanation (SHAP) model (Lundberg and Lee, 2017). These feature selection technologies could present the contribution of channels and their relationships, thus optimizing the model by selecting the more important features.

## Results

### Behavior Results

Repeated ANOVA of accuracy showed that there were no significant differences in scarcity mindset [*F*(1,26) = 1.349, *p* = 0.256, $\eta_{p}^{2}=0.049$], reward conditions [*F*(1,26) = 2.232, *p* = 0.147, $\eta_{p}^{2}=0.079$], and interaction effect [*F*(1,26) = 1.537, *p* = 0.226, $\eta_{p}^{2}=0.056$]. rANOVA of reaction time showed no significant differences in perceived scarcity [*F*(1,26) = 0.215, *p* = 0.647, $\eta_{p}^{2}=0.008$], reward conditions [*F*(1,26) = 1.722, *p* = 0.201, $\eta_{p}^{2}=0.062$], and interaction effect [*F*(1,26) = 0.032, *p* = 0.859, $\eta_{p}^{2}=0.001$]. The simple effect analysis results showed no significant difference under abundant resources [*F*(1,26) = 0.091, *p* = 0.765, $\eta_{p}^{2}=0.003$]. While a weakly significant effect was found under the condition of insufficient resources, the accuracy of the 10 yuan reward (*M* = 0.68, SD = 0.06) was higher [*F*(1,26) = 3.826, *p* = 0.061, $\eta_{p}^{2}=0.128$] than that of 0 yuan reward (*M* = 0.63, SD = 0.07) weakly. The accuracy and reaction time results are shown in Figure 1B.

### Functional Near-Infrared Spectroscopy Results

The descriptive statistical results of functional connectivity were shown in Figure 2C after taking absolute values. The results showed that some correlation coefficient was high in the task, but the overall correlation was moderate.

The results of rANOVA after FDR showed that channel 2 and channel 16 had a significant difference in the scarcity effect of functional connectivity, Abundance mindset (*M* = 0.699, SD = 0.286) was significantly greater [*F*(1,24) = 20.278, *p* < 0.000, $\eta_{p}^{2}=0.458$] than that of scarcity mindset (*M* = 0.459, SD = 0.500), shown in Figure 2B.

Table 1 and Figure 2D described the ROI locations of each channel and the statistical test results. The difference in the maximum activation of total-Hb was mainly seen in the left hemisphere (channels 1, 3, 4, and 6), with a slight difference in the right hemisphere (channel 17) and in channel 12 between R-DLPFC and L-DLPFC.

### Tree-Based Model Results

As shown in Table 2, combining with the precision, recall rate, F1 index of the test dataset, as well as the ROC curve and AUC of each model (Figure 1C), the Random Forest model had the best performance. The model results were provided by the Scikit-Learning software package based on Python 3.8.5 (Pedregosa et al., 2011). The precision can reach about 63%, while F1, recall rate, and accuracy are above 60% in the Random Forest model. The result showed that the classification model established by the Random Forest model was acceptable.

**TABLE 2:** Precision, recall, and F1 in training and testing procession in tree-based model.

Test precession	Class	Precision	Recall	F1	Support
RFC testing	Reward	0.63	0.62	0.62	598
Accuracy = 0.63	No reward	0.63	0.64	0.63	602
	Macro	0.63	0.63	0.63	
	Weighted	0.63	0.63	0.63	
CatBoost testing	Reward	0.62	0.60	0.61	598
Accuracy = 0.62	No reward	0.62	0.64	0.63	602
	Macro	0.62	0.62	0.62	
	Weighted	0.62	0.62	0.62	
AdaBoost testing	Reward	0.57	0.55	0.56	598
Accuracy = 0.57	No reward	0.57	0.58	0.58	602
	Macro	0.57	0.57	0.57	
	Weighted	0.57	0.57	0.57	
XGBoost testing	Reward	0.61	0.58	0.60	598
Accuracy = 0.61	No reward	0.60	0.63	0.62	602
	Macro	0.61	0.61	0.61	
	Weighted	0.61	0.61	0.61	
LightBoost testing	Reward	0.63	0.61	0.62	598
Accuracy = 0.62	No reward	0.62	0.64	0.63	602
	Macro	0.62	0.62	0.62	
	Weighted	0.62	0.62	0.62	

**FIGURE 4:**
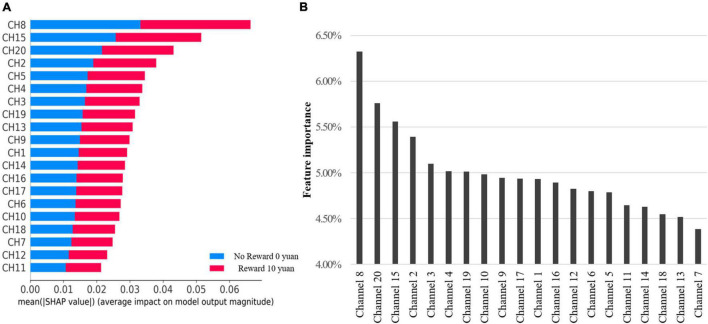
**(A)** It shows the average absolute SHAP value (impact) of each channel on model output magnitude. **(B)** The feature importance of each channel by RFC model directly.

According to the test based on the Gini Index in Figure 4A and SHAP in Figure 4B results, the contribution of channel 8 ranks first in both. Channels 20, 15, 2 are included in top 4. Others are unstable in Random Forest and SHAP models. Accordingly, we selected top 4 features to simplify the model.

**FIGURE 5:**
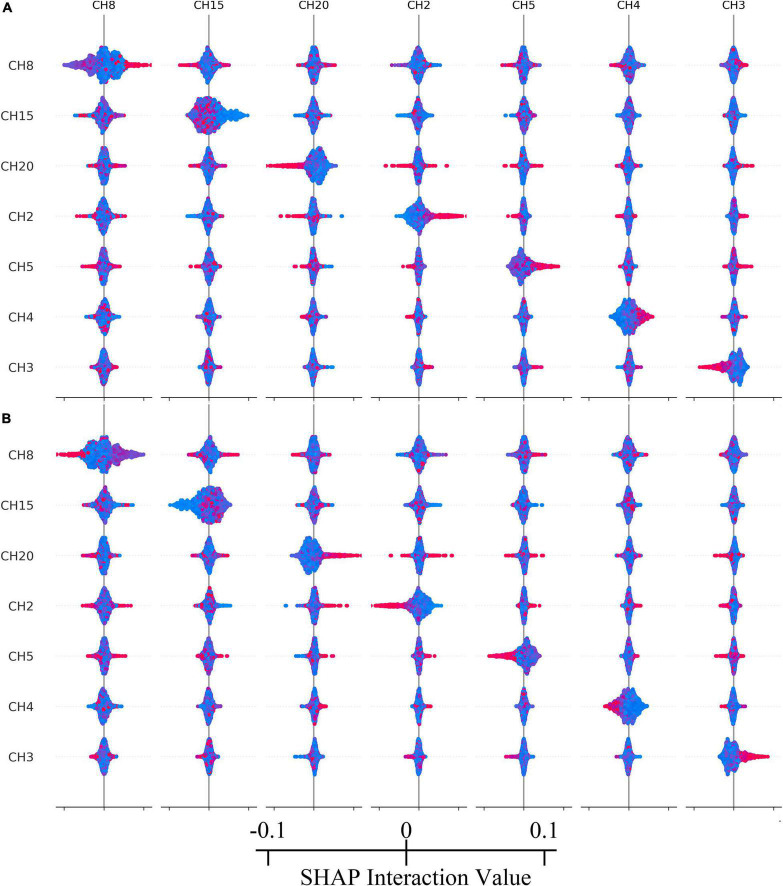
**(A)** The interaction relationship within top-7 important channels of reward condition. **(B)** The interaction relationship within top-7 important channels of no reward condition. Colors represent eigenvalues, red is high, blue is low.

According to calculating the SHAP value interactive analysis, it was conducted on the top 7 contribution ranking features. See Figure 5 for the results.

## Discussion

This study was the first to investigate the degree of scarcity mindset in the MID task, aiming to find the brain activation mechanism of the scarcity mindset and verify the neural mechanism of reward in fNIRS. MID tasks adjusted the accuracy to 66% based on the overall performance. However, this study still found that reward can improve the accuracy of performance with a scarcity mindset during autoadaptive processing. In the left PFC, brain activation increased during the reward condition, and the functional connectivity between the left and right PFC diminished during the scarcity mindset.

### Effects of the Scarcity Mindset

According to the present study’s results, the reward improved the accuracy with the scarcity mindset and did not change it with the abundance mindset. Moreover, the neural synchronization between channels 2 (L-VLPFC) and 16 (R-DLPFC) was impeded by a scarcity mindset. An attentional mechanism is a mechanism of cognitive or behavioral disorders caused by scarcity mindset conditions (Tomm and Zhao, 2018). When subjects felt scarcity, their cognition and behavior were impeded by the attentional system, and their neural synchronization in the PFC was decreased. This can be explained by the excessive psychological compensation mechanism (Bäckman and Dixon, 1992). When the subjects’ scarcity mindset was activated, the concrete embodiment formed an unsafe inner feeling and then chased the reward to balance the insecurity. However, this kind of compensation was excessive, in the end, because the attention resource focused on the superficial (Tomm and Zhao, 2018) and hindered the completion of the task.

According to prospect theory (Kahneman and Tversky, 1979; Wakker, 2010; Tversky and Kahneman, 2016), in this study, if we assume that the initial amount of money given is the reference point and the reward per round was based on the relative potency of the reference point, we can find that a stronger brain connection is activated in the PFC, which is a relative potency change. This result was consistent with the EEG findings (Xiang et al., 2009). In the insufficient-insufficient condition, the correlation between the 10-yuan reward and 0-yuan reward was higher than that in the sufficient-resource condition. In combination with the previous hypothesis, it was found that a high relative potency led to more robust activation of brain connections in the PFC, which the prospect theory could explain. Under different scarcity mindsets, subjects made their own decisions using relative potency, with reference points to compare the initial funding with each round of rewards. The activation of reward expectations in the L-VLPFC was found, and a connection difference between the L-DLPFC and R-DLPFC was also found under different scarcity mindsets. R-DLPFC did not activate the expectation of reward. Therefore, we surmised that R-DLPFC was only more sensitive to the sense of scarcity caused by high relative potency, i.e., a scarcity mindset, and was not sensitive to low relative potency, i.e., without a scarcity mindset. There may be a reverse relationship between the R-DLPFC and the correlation efficiency value. The L-VLPFC has different responses to various associative effects and is sensitive to reward processing.

### Brain Activity to Reward

The DLPFC and PFC are very important cortices in the human brain that participate in high-level cognitive activities, such as planning, decision-making, and comparison. According to previous fMRI results, the DLPFC was also involved in reward processing (Ballard et al., 2011). This study showed that the activation of LDLPFC (channel 6) in the reward condition was significantly greater than that in the non-reward condition, similar to previous studies’ results. fNIRS was also involved in reward processing in the DLPFC. Multiple ventrolateral prefrontal cortex (VLPFC) parts (L-VLPFC: channels 1, 3, and 4 and R-VLPFC: channel 17) were found to be activated by reward conditions. Reward activation of the VLPFC can be explained by polymorphisms of the serotonin receptor gene (Nomura et al., 2007). Additionally, reward-sensitive individuals have been found to have increased connectivity between the posterior cingulate and anterior central gyrus of the VLPFC (Cho et al., 2016). Simultaneously, an fMRI study found a significant positive correlation between high sensation-seeking reward expectations and higher L-VLPFC activation (Edmiston et al., 2020). In the MID task, subjects tended to reward and expected to be rewarded based on activated expectations, which activated the VLPFC. This study directly showed that the expectation of reward did indeed lead to significant activation of the VLPFC.

### Classification to Reward Function

According to the machine learning model’s classification results, channel 8 (located in the frontal pole) contributed the most, but the rANOVA results showed that channel 8 did not indicate a significant difference in reward conditions. This result was reasonable because rANOVA was not good at higher-order interactions and assumed that the channels were independent rather than acting together. However, the tree-based classification model was different, considering 20 channel interactions and integrating all interactions to predict the classification tags. Therefore, the results of this study were in line with expectations. At the same time, in the interaction test of the SHAP model, channels 8, 15, 20, 2, 5, 4, and 3 all showed different degrees of interaction, which also confirmed that reward processing was carried out jointly by various brain regions, rather than independently, and that there was a high-order interaction.

This study found that prospect theory and its reference points have involved few studies in neuroscience. Based on the current study, it is possible to determine individual reference time points, but most previous studies were limited to behavioral experiments. In this study, the relative reference points affect the relative potency of the scarcity mindset in a block because of the block design. Therefore, this study inferred that the relative reference point affected the whole task rather than a single point in time, although this is only a hypothesis at present and will be tested in future studies.

### Limitations

In reviewing this study, there was only marginal significance in the behavioral experiment because we did not have enough subjects in our study. The effect size ($\eta_{p}^{2}<0.2$) of behavior determined that our study was not a large effect size experiment, and it is reasonable that no simple effect was found. However, the results still reveal that the PFC can be affected by a combination of scarcity and reward when setting relative reference points. Furthermore, a study using MID to study reward processing combined with fMRI and EEG found that there was no difference in fMRI-recorded images between reward and loss conditions measured directly (Pfabigan et al., 2014). This study, therefore, ventured to speculate that it might be possible to explore neural and blood oxygen changes in the PFC caused by reward conditions by setting relative potency as the reference point.

It’s a burgeoning field that employs machine learning approaches to decode individual differences in behavioral phenotypes from brain imaging data. The application of machine learning methods to predictive modeling in neuroimaging is relatively modest due to this field is still immature. In most neuroimaging-based studies focusing on the prediction problem, the overall accuracy was highlight as the ultimate model performance measure. Over the past few years, many researchers have proposed their accuracy are higher than other studies, this opinion is inappropriate by simply compared the percentage. Without well-matched study design and variables (i.e., sample size, age, sex, parameters, data modality, ROI, features of interest, feature selection methods, classifier type, and CV scheme), there is no comparison among these different studies (Rashid and Calhoun, 2020). More importantly, for this study, it is not our goal to optimize the algorithm to achieve a very high accuracy rate. We hope to find an algorithm that is sensitive to brain blood oxygen activation signal by comparing these algorithms and provide a visual model for decoding individual differences.

In addition, machine learning techniques require a large amount of training data to identify more generalized features and improved performance, and neuroimaging studies in general have very small sample size (usually less than 50 subjects) (Rashid and Calhoun, 2020). The small sample size may introduce poorer performance. And that, Yang et al. (2016) used SVM classifier to compare unimodal versus multi modal accuracy and showed that multimodal features achieved higher accuracy (77.91%) than single modality accuracy (72.09%), also several other studies found that integrating multimodal data improved prediction accuracy (Qureshi et al., 2017; Yang et al., 2018; Jiang et al., 2020). Therefore, lots of efforts on big data, multi-modal fusion approaches and advanced machine learning techniques are required in future studies.

## Conclusion

In conclusion, our results provide novel experimental evidence that behavioral performance and bold activity in the PFC can explain scarcity and reward mechanisms. The neural synchronization between the left and right PFC, a main cognitive control region, decreased when individuals suffered from the scarcity condition. The left PFC hemisphere, mostly the L-VLPFC and L-DLPFC, was activated in reward conditions, and it can be detected by the tree-based machine learning model, especially the random forest model SHAP model, to indicate a high-level interrelationship within the PFC. To the best of our knowledge, this is the first study employing a tree-based machine learning model analysis of the scarcity mindset and reward. This study provides directions for high-level interrelationship analysis of the scarcity mindset and reward or possibly other types of brain functions.

## Data Availability Statement

The raw data supporting the conclusions of this article will be made available by the authors, without undue reservation.

## Ethics Statement

The studies involving human participants were reviewed and approved by the Henan University. The patients/participants provided their written informed consent to participate in this study.

## Author Contributions

XJ contributed to design and analysis the experiment and data. CZ, NA, WG, and JL collected the data. XJ and YC drafted the manuscript. XJ, CZ, and YC revised the manuscript. All authors contributed to the article and approved the submitted version.

## Conflict of Interest

The authors declare that the research was conducted in the absence of any commercial or financial relationships that could be construed as a potential conflict of interest.

## Publisher’s Note

All claims expressed in this article are solely those of the authors and do not necessarily represent those of their affiliated organizations, or those of the publisher, the editors and the reviewers. Any product that may be evaluated in this article, or claim that may be made by its manufacturer, is not guaranteed or endorsed by the publisher.
